# Supplementary material for: Associations between multiple long-term conditions and mortality in diverse ethnic groups
Source: PLoS One. 2022 Apr 1;17(4):e0266418. doi: 10.1371/journal.pone.0266418 (PMC8974956; doi:10.1371/journal.pone.0266418)
Supplement: S1 Table — (DOCX) [file pone.0266418.s001.docx]

**S1 Table. Long-term conditions counted in the current study**

| **Long-term condition** | **Body system** | **Time criteria (ever if not specified)** |  |
| --- | --- | --- | --- |
| Cancer | Cancers | In last 5 years |  |
| Atrial fibrillation | Diseases of the Circulatory System |  |  |
| Heart disease | Diseases of the Circulatory System |  |  |
| Heart failure | Diseases of the Circulatory System |  |  |
| Hypertension | Diseases of the Circulatory System |  |  |
| Stroke | Diseases of the Circulatory System |  | stroke |
| Vascular disease | Diseases of the Circulatory System |  |  |
| Diverticulosis | Diseases of the Digestive System |  |  |
| IBS | Diseases of the Digestive System |  |  |
| Liver disease | Diseases of the Digestive System |  |  |
| Hearing loss | Diseases of the Ear |  |  |
| Blindness | Diseases of the Eye |  |  |
| Diabetes | Diseases of the Endocrine System |  |  |
| Thyroid disorders | Diseases of the Endocrine System |  |  |
| Kidney disease | Diseases of the Genitourinary System |  |  |
| Asthma | Diseases of the Respiratory System | In last 12 months |  |
| Bronchiectasis | Diseases of the Respiratory System |  |  |
| COPD | Diseases of the Respiratory System |  |  |
| Viral hepatitis | Infectious Diseases |  |  |
| Alcohol misuse | Mental Health Disorders |  |  |
| Anxiety/depression | Mental Health Disorders | In last 12 months |  |
| Anorexia/bulimia | Mental Health Disorders |  |  |
| Dementia | Mental Health Disorders |  |  |
| Learning disability | Mental Health Disorders |  |  |
| Schizophrenia | Mental Health Disorders |  |  |
| Substance misuse | Mental Health Disorders |  |  |
| Arthritis | Musculoskeletal conditions |  |  |
| Epilepsy | Neurological conditions |  | epilepsy |
| Migraine | Neurological conditions |  |  |
| Multiple sclerosis | Neurological conditions |  |  |
| Parkinsons disease | Neurological conditions |  |  |
| Psoriasis | Skin conditions |  |  |
