# Supplementary material for: Associations between multiple long-term conditions and mortality in diverse ethnic groups
Source: PLoS One. 2022 Apr 1;17(4):e0266418. doi: 10.1371/journal.pone.0266418 (PMC8974956; doi:10.1371/journal.pone.0266418)
Supplement: S2 Table — (DOCX) [file pone.0266418.s002.docx]

**S2 Table. Characteristics of analytical sample by ethnicity**

|  | Patients excluded due to missing data (% of patients) | Total analytical sample  (% of patients) | Bangla-deshi  N (%) | Pakistani  N (%) | Indian  N (%) | Other Asian  N (%) | Chinese  N (%) | Black African  N (%) | Black Caribbean  N (%) | Other Black  N (%) | Mixed  N (%) | Other  N (%) | White  N (%) |
| --- | --- | --- | --- | --- | --- | --- | --- | --- | --- | --- | --- | --- | --- |
| n | 67941 | 532059 | 3711 | 9233 | 15142 | 9022 | 4608 | 11517 | 7357 | 3119 | 7227 | 6910 | 454213 |
| Age 18-29y | 23.2 | 15.7 | 1008 (27.2) | 2312 (25.0) | 2518 (16.6) | 1964 (21.8) | 1903 (41.3) | 2292 (19.9) | 1061 (14.4) | 849 (27.2) | 1988 (27.5) | 1496 (21.6) | 66258 (14.6) |
| Age 30-39y | 15.9 | 17.2 | 1183 (31.9) | 2786 (30.2) | 4286 (28.3) | 2379 (26.4) | 1000 (21.7) | 2935 (25.5) | 1233 (16.8) | 780 (25.0) | 1905 (26.4) | 1928 (27.9) | 71244 (15.7) |
| Age 40-49y | 19.6 | 17.7 | 826 (22.3) | 1976 (21.4) | 2943 (19.4) | 2131 (23.6) | 715 (15.5) | 3140 (27.3) | 1348 (18.3) | 632 (20.3) | 1474 (20.4) | 1628 (23.6) | 77603 (17.1) |
| Age 50-59y | 19.8 | 17.2 | 312 (8.4) | 978 (10.6) | 2174 (14.4) | 1210 (13.4) | 446 (9.7) | 1985 (17.2) | 1783 (24.2) | 560 (18.0) | 1050 (14.5) | 975 (14.1) | 80011 (17.6) |
| Age 60-69y | 12.3 | 13.9 | 199 (5.4) | 681 (7.4) | 1753 (11.6) | 799 (8.9) | 305 (6.6) | 673 (5.8) | 724 (9.8) | 137 (4.4) | 436 (6.0) | 475 (6.9) | 67523 (14.9) |
| Age 70-79y | 5.9 | 10.5 | 123 (3.3) | 319 (3.5) | 937 (6.2) | 380 (4.2) | 140 (3.0) | 374 (3.2) | 661 (9.0) | 97 (3.1) | 240 (3.3) | 266 (3.8) | 52338 (11.5) |
| Age 80+y | 3.4 | 7.8 | 60 (1.6) | 181 (2.0) | 531 (3.5) | 159 (1.8) | 99 (2.1) | 118 (1.0) | 547 (7.4) | 64 (2.1) | 134 (1.9) | 142 (2.1) | 39236 (8.6) |
| Women | 35.6 | 51.9 | 1697 (45.7) | 4259 (46.1) | 7331 (48.4) | 4463 (49.5) | 2446 (53.1) | 5897 (51.2) | 3979 (54.1) | 1578 (50.6) | 3880 (53.7) | 3335 (48.3) | 237282 (52.2) |
| IMD 1 (least deprived) | 15.0 | 10.7 | 71 (1.9) | 291 (3.2) | 1130 (7.5) | 567 (6.3) | 335 (7.3) | 180 (1.6) | 112 (1.5) | 64 (2.1) | 449 (6.2) | 406 (5.9) | 53518 (11.8) |
| IMD 2 | 12.5 | 10.3 | 65 (1.8) | 299 (3.2) | 1126 (7.4) | 595 (6.6) | 379 (8.2) | 227 (2.0) | 139 (1.9) | 84 (2.7) | 421 (5.8) | 381 (5.5) | 51305 (11.3) |
| IMD 3 | 11.9 | 10.5 | 113 (3.0) | 399 (4.3) | 1174 (7.8) | 826 (9.2) | 422 (9.2) | 312 (2.7) | 168 (2.3) | 125 (4.0) | 500 (6.9) | 494 (7.1) | 51265 (11.3) |
| IMD 4 | 10.6 | 10.0 | 107 (2.9) | 448 (4.9) | 1234 (8.1) | 739 (8.2) | 395 (8.6) | 430 (3.7) | 270 (3.7) | 118 (3.8) | 463 (6.4) | 456 (6.6) | 48316 (10.6) |
| IMD 5 | 9.5 | 9.6 | 112 (3.0) | 594 (6.4) | 1451 (9.6) | 832 (9.2) | 336 (7.3) | 562 (4.9) | 410 (5.6) | 203 (6.5) | 604 (8.4) | 585 (8.5) | 45523 (10.0) |
| IMD 6 | 8.9 | 9.9 | 232 (6.3) | 863 (9.3) | 1907 (12.6) | 1048 (11.6) | 488 (10.6) | 943 (8.2) | 724 (9.8) | 259 (8.3) | 705 (9.8) | 596 (8.6) | 44964 (9.9) |
| IMD 7 | 9.9 | 10.4 | 321 (8.6) | 1128 (12.2) | 1922 (12.7) | 1286 (14.3) | 626 (13.6) | 1317 (11.4) | 980 (13.3) | 372 (11.9) | 862 (11.9) | 897 (13.0) | 45448 (10.0) |
| IMD 8 | 7.6 | 9.7 | 751 (20.2) | 1410 (15.3) | 2210 (14.6) | 1377 (15.3) | 667 (14.5) | 2268 (19.7) | 1325 (18.0) | 547 (17.5) | 1051 (14.5) | 1178 (17.0) | 38932 (8.6) |
| IMD 9 | 7.6 | 10.1 | 993 (26.8) | 1733 (18.8) | 1726 (11.4) | 1104 (12.2) | 573 (12.4) | 3188 (27.7) | 1753 (23.8) | 692 (22.2) | 1200 (16.6) | 1088 (15.7) | 39877 (8.8) |
| IMD 10 | 6.7 | 8.7 | 946 (25.5) | 2068 (22.4) | 1262 (8.3) | 648 (7.2) | 387 (8.4) | 2090 (18.1) | 1476 (20.1) | 655 (21.0) | 972 (13.4) | 829 (12.0) | 35065 (7.7) |
| Complex multi-morbidity | 3.3 | 11.1 | 228 (6.1) | 679 (7.4) | 1233 (8.1) | 442 (4.9) | 117 (2.5) | 437 (3.8) | 848 (11.5) | 164 (5.3) | 326 (4.5) | 267 (3.9) | 54332 (12.0) |
| Baseline number of conditions; mean (SD) | 0.53 (0.97) | 1.16 (1.49) | 0.72 (1.17) | 0.82 (1.29) | 0.88 (1.32) | 0.63 (1.09) | 0.33 (0.81) | 0.57 (0.95) | 1.11 (1.42) | 0.63 (1.02) | 0.67 (1.05) | 0.54 (1.00) | 1.23 (1.52) |
| Died during follow-up |  |  | 54 (1.5) | 184 (2.0) | 398 (2.6) | 132 (1.5) | 54 (1.2) | 135 (1.2) | 350 (4.8) | 46 (1.5) | 111 (1.5) | 104 (1.5) | 29892 (6.6) |
| Follow-up time in years; mean (SD) |  |  | 4.31 (1.41) | 4.31 (1.39) | 4.20 (1.48) | 4.03 (1.63) | 3.47 (1.90) | 4.05 (1.59) | 4.31 (1.36) | 4.16 (1.50) | 4.06 (1.59) | 4.00 (1.63) | 4.25 (1.43) |
| Number of conditions at end of follow-up; mean (SD) |  |  | 0.96 (1.39) | 1.07 (1.52) | 1.12 (1.55) | 0.83 (1.31) | 0.41 (0.97) | 0.74 (1.13) | 1.40 (1.67) | 0.83 (1.22) | 0.82 (1.24) | 0.71 (1.20) | 1.55 (1.79) |

IMD Index of Multiple Deprivation in local area
