# Supplementary material for: Associations between multiple long-term conditions and mortality in diverse ethnic groups
Source: PLoS One. 2022 Apr 1;17(4):e0266418. doi: 10.1371/journal.pone.0266418 (PMC8974956; doi:10.1371/journal.pone.0266418)
Supplement: S3 Table — (DOCX) [file pone.0266418.s003.docx]

**S3 Table. Number of conditions and complex multimorbidity prevalence by age and ethnicity**

|  | Bangla-deshi | Pakistani  N (%) | Indian  N (%) | Other Asian  N (%) | Chinese  N (%) | Black African  N (%) | Black Caribbean  N (%) | Black Other  N (%) | Mixed  N (%) | Other  N (%) | White  N (%) |
| --- | --- | --- | --- | --- | --- | --- | --- | --- | --- | --- | --- |
| n | 3711 | 9233 | 15142 | 9022 | 4608 | 11517 | 7357 | 3119 | 7227 | 6910 | 454213 |
| Age 18-49y  Number of conditions; mean  Complex multimorbidity; % | 0.4  1.4 | 0.5  1.8 | 0.4  1.2 | 0.3  1.0 | 0.1  0.4 | 0.3  1.0 | 0.5  1.7 | 0.4  1.1 | 0.4  1.1 | 0.3  0.7 | 0.6  2.2 |
| Age 50-69y  Number of conditions; mean  Complex multimorbidity; % | 1.9  25.6 | 1.9  23.1 | 1.6  17.6 | 1.3  12.7 | 0.9  7.7 | 1.2  10.3 | 1.4  15.6 | 1.1  13.0 | 1.2  11.6 | 1.1  10.4 | 1.5  15.1 |
| Age 70+y  Number of conditions; mean  Complex multimorbidity; % | 2.9  40.0 | 3.4  51.4 | 3.2  50.1 | 3.0  45.9 | 2.3  35.4 | 2.3  35.6 | 3.2  53.4 | 2.9  54.7 | 2.8  49.3 | 2.5  36.6 | 3.2  49.5 |
