# Supplementary material for: Associations between multiple long-term conditions and mortality in diverse ethnic groups
Source: PLoS One. 2022 Apr 1;17(4):e0266418. doi: 10.1371/journal.pone.0266418 (PMC8974956; doi:10.1371/journal.pone.0266418)
Supplement: S4 Table — Model 1 for constant association between number of conditions and mortality risk same across ethnic groups; Model 2 including interaction between ethnicity and number of conditions; Model 3 including possible explanatory factors. (DOCX) [file pone.0266418.s004.docx]

**S4 Table. Cox regression estimates for models including number of conditions.**

|  | Model 1 | |  | | |  | | Model 2 | | |  | |  | | | | Model 3 | |  | | |  |
| --- | --- | --- | --- | --- | --- | --- | --- | --- | --- | --- | --- | --- | --- | --- | --- | --- | --- | --- | --- | --- | --- | --- |
| Covariate | Regression estimate | Standard error | | p-value | | | Regression estimate | | Standard error | | | p-value | | Regression estimate | | Standard error | | p-value | | |  |  |
| Women | -0.28 | 0.01 | | p<0.001 | | | -0.28 | | 0.01 | | | p<0.001 | | -0.29 | | 0.01 | | p<0.001 | | |  |  |
| Baseline age | 0.10 | 0.00 | | p<0.001 | | | 0.10 | | 0.001 | | | p<0.001 | | 0.10 | | 0.001 | | p<0.001 | | |  |  |
| Baseline number of conditions | 0.29 | 0.01 | | p<0.001 | | | 0.29 | | 0.01 | | | p<0.001 | | 0.28 | | 0.01 | | p<0.001 | | |  |  |
| Age x number of conditions | -0.003 | 0.000 | | p<0.001 | | | -0.003 | | 0.0003 | | | p<0.001 | | -0.003 | | 0.0003 | | p<0.001 | | |  |  |
| Ethnicity (main effect) |  |  | |  | | |  | |  | | |  | |  | |  | |  | | |  |  |
| Bangladeshi | -0.16 | 0.22 | | p=0.4 | | | -0.10 | | 0.22 | | | p=0.7 | | -0.28 | | 0.25 | | p=0.2 | | |  |  |
| Pakistani | 0.08 | 0.13 | | p=0.6 | | | -0.26 | | 0.15 | | | p=0.2 | | -0.39 | | 0.15 | | p=0.01 | | |  |  |
| Indian | -0.48 | 0.12 | | p<0.001 | | | -0.54 | | 0.12 | | | p<0.001 | | -0.57 | | 0.12 | | p<0.001 | | |  |  |
| Other Asian | -0.30 | 0.16 | | p=0.06 | | | -0.41 | | 0.16 | | | p=0.009 | | -0.45 | | 0.16 | | p=0.003 | | |  |  |
| Chinese | -0.90 | 0.32 | | p=0.004 | | | -0.98 | | 0.33 | | | p=0.002 | | -0.98 | | 0.33 | | p=0.001 | | |  |  |
| Black African | -0.03 | 0.12 | | p=0.8 | | | -0.18 | | 0.14 | | | p=0.2 | | -0.34 | | 0.14 | | p=0.01 | | |  |  |
| Black Caribbean | 0.19 | 0.13 | | p=0.1 | | | 0.08 | | 0.12 | | | p=0.5 | | -0.04 | | 0.12 | | p=0.7 | | |  |  |
| Other Black | 0.27 | 0.20 | | p=0.2 | | | -0.14 | | 0.23 | | | p=0.5 | | -0.28 | | 0.24 | | p=0.2 | | |  |  |
| Mixed | 0.20 | 0.13 | | p=0.1 | | | 0.11 | | 0.16 | | | p=0.5 | | 0.02 | | 0.16 | | p=0.9 | | |  |  |
| Other | -0.24 | 0.19 | | p=0.2 | | | -0.37 | | 0.19 | | | p=0.06 | | -0.45 | | 0.19 | | p=0.03 | | |  |  |
| Age x ethnicity interaction: |  |  | |  | | |  | |  | | |  | |  | |  | |  | | |  |  |
| Bangladeshi | -0.004 | 0.009 | | p=0.7 | | | -0.002 | | 0.010 | | | p=0.8 | | -0.003 | | 0.010 | | p=0.8 | | |  |  |
| Pakistani | -0.009 | 0.005 | | p=0.09 | | | -0.019 | | 0.006 | | | p=0.006 | | -0.019 | | 0.006 | | p=0.002 | | |  |  |
| Indian | 0.007 | 0.004 | | p=0.1 | | | 0.005 | | 0.004 | | | p=0.3 | | 0.004 | | 0.005 | | p=0.4 | | |  |  |
| Other Asian | -0.005 | 0.007 | | p=0.5 | | | -0.010 | | 0.007 | | | p=0.2 | | -0.010 | | 0.008 | | p=0.2 | | |  |  |
| Chinese | 0.019 | 0.011 | | p=0.07 | | | 0.015 | | 0.012 | | | p=0.2 | | 0.014 | | 0.011 | | p=0.3 | | |  |  |
| Black African | -0.015 | 0.007 | | p=0.02 | | | -0.021 | | 0.006 | | | p=0.003 | | -0.021 | | 0.006 | | p=0.003 | | |  |  |
| Black Caribbean | -0.014 | 0.005 | | p=0.002 | | | -0.018 | | 0.004 | | | p<0.001 | | -0.019 | | 0.004 | | p<0.001 | | |  |  |
| Other Black | -0.030 | 0.010 | | p=0.003 | | | -0.045 | | 0.011 | | | p<0.001 | | -0.044 | | 0.011 | | p<0.001 | | |  |  |
| Mixed | -0.024 | 0.006 | | p<0.001 | | | -0.027 | | 0.007 | | | p<0.001 | | -0.027 | | 0.008 | | p<0.001 | | |  |  |
| Other | -0.002 | 0.008 | | p=0.8 | | | -0.006 | | 0.007 | | | p=0.4 | | -0.006 | | 0.008 | | p=0.5 | | |  |  |
| Number of conditions x ethnicity interaction: |  |  | |  | | |  | |  | | |  | |  | |  | |  | | |  |  |
| Bangladeshi |  |  | |  | | | -0.03 | | 0.07 | | | p=0.7 | | -0.02 | | 0.07 | | p=0.8 | | |  |  |
| Pakistani |  |  | |  | | | 0.15 | | 0.04 | | | p<0.001 | | 0.16 | | 0.04 | | p<0.001 | | |  |  |
| Indian |  |  | |  | | | 0.04 | | 0.03 | | | p=0.1 | | 0.04 | | 0.03 | | p=0.1 | | |  |  |
| Other Asian |  |  | |  | | | 0.08 | | 0.05 | | | p=0.08 | | 0.09 | | 0.05 | | p=0.06 | | |  |  |
| Chinese |  |  | |  | | | 0.07 | | 0.08 | | | p=0.4 | | 0.07 | | 0.08 | | p=0.4 | | |  |  |
| Black African |  |  | |  | | | 0.11 | | 0.05 | | | p=0.03 | | 0.11 | | 0.05 | | p=0.02 | | |  |  |
| Black Caribbean |  |  | |  | | | 0.07 | | 0.03 | | | p=0.01 | | 0.07 | | 0.03 | | p=0.009 | | |  |  |
| Other Black |  |  | |  | | | 0.27 | | 0.08 | | | p=0.006 | | 0.27 | | 0.08 | | p=0.005 | | |  |  |
| Mixed |  |  | |  | | | 0.07 | | 0.08 | | | p=0.4 | | 0.06 | | 0.06 | | p=0.4 | | |  |  |
| Other |  |  | |  | | | 0.10 | | 0.06 | | | p=0.05 | | 0.10 | | 0.06 | | p=0.07 | | |  |  |
| Number of conditions at end of follow-up |  |  | |  | | |  | |  | | |  | | 0.005 | | 0.005 | | p=0.5 | | |  |  |
| Index of Multiple Deprivation decile |  |  | |  | | |  | |  | | |  | |  | |  | |  | | |  |  |
| 2 |  |  | |  | | |  | |  | | |  | | 0.11 | | 0.02 | | p<0.001 | | |  |  |
| 3 |  |  | |  | | |  | |  | | |  | | 0.15 | | 0.02 | | p<0.001 | | |  |  |
| 4 |  |  | |  | | |  | |  | | |  | | 0.17 | | 0.03 | | p<0.001 | | |  |  |
| 5 |  |  | |  | | |  | |  | | |  | | 0.17 | | 0.03 | | p<0.001 | | |  |  |
| 6 |  |  | |  | | |  | |  | | |  | | 0.20 | | 0.03 | | p<0.001 | | |  |  |
| 7 |  |  | |  | | |  | |  | | |  | | 0.29 | | 0.03 | | p<0.001 | | |  |  |
| 8 |  |  | |  | | |  | |  | | |  | | 0.36 | | 0.03 | | p<0.001 | | |  |  |
| 9 |  |  | |  | | |  | |  | | |  | | 0.44 | | 0.03 | | p<0.001 | | |  |  |
| 10 |  |  | |  | | |  | |  | | |  | | 0.56 | | 0.03 | | p<0.001 | | |  |  |
| Likelihood ratio test |  | | | | 21.8 on 10 df, 0.05>p>0.01 compared with model 1 | | | | |  | | | | | 392 on 10 df, p<0.001 compared with model 2 | | | | |  |  |  |

Model 1 for constant association between number of conditions and mortality risk same across ethnic groups; Model 2 including interaction between ethnicity and number of conditions; Model 3 including possible explanatory factors
