# Supplementary material for: Associations between multiple long-term conditions and mortality in diverse ethnic groups
Source: PLoS One. 2022 Apr 1;17(4):e0266418. doi: 10.1371/journal.pone.0266418 (PMC8974956; doi:10.1371/journal.pone.0266418)
Supplement: S5 Table — (DOCX) [file pone.0266418.s005.docx]

**S5 Table. Cox regression estimates for model including complex multimorbidity**

| Covariate | Regression estimate | Standard error | p-value |
| --- | --- | --- | --- |
| Women | -0.28 | 0.01 | p<0.001 |
| Baseline age | 0.10 | 0.001 | p<0.001 |
| Baseline complex multimorbidity | 0.85 | 0.03 | p<0.001 |
| Age x complex multimorbidity | -0.01 | 0.001 | p<0.001 |
| Ethnicity (main effect) |  |  |  |
| Bangladeshi | -0.18 | 0.21 | p=0.4 |
| Pakistani | -0.06 | 0.14 | p=0.7 |
| Indian | -0.48 | 0.11 | p<0.001 |
| Other Asian | -0.40 | 0.15 | p=0.01 |
| Chinese | -1.05 | 0.30 | p<0.001 |
| Black African | -0.20 | 0.13 | p=0.1 |
| Black Caribbean | 0.14 | 0.12 | p=0.3 |
| Other Black | -0.11 | 0.22 | p=0.6 |
| Mixed | 0.16 | 0.13 | p=0.2 |
| Other | -0.37 | 0.19 | p=0.05 |
| Age x ethnicity interaction: |  |  |  |
| Bangladeshi | -0.003 | 0.009 | p=0.8 |
| Pakistani | -0.013 | 0.006 | p=0.02 |
| Indian | 0.007 | 0.004 | p=0.1 |
| Other Asian | -0.006 | 0.008 | p=0.5 |
| Chinese | 0.012 | 0.011 | p=0.3 |
| Black African | -0.018 | 0.007 | p=0.008 |
| Black Caribbean | -0.014 | 0.005 | p=0.003 |
| Other Black | -0.045 | 0.011 | p<0.001 |
| Mixed | -0.020 | 0.007 | p=0.004 |
| Other | -0.002 | 0.008 | p=0.8 |
| Complex multimorbidity x ethnicity interaction: |  |  |  |
| Bangladeshi | 0.01 | 0.28 | p=0.9 |
| Pakistani | 0.45 | 0.16 | p=0.004 |
| Indian | 0.01 | 0.11 | p=0.9 |
| Other Asian | 0.23 | 0.21 | p=0.3 |
| Chinese | 0.62 | 0.30 | p=0.04 |
| Black African | 0.46 | 0.17 | p=0.009 |
| Black Caribbean | 0.06 | 0.10 | p=0.6 |
| Other Black | 1.15 | 0.33 | p<0.001 |
| Mixed | -0.22 | 0.25 | p=0.4 |
| Other | 0.21 | 0.21 | p=0.3 |
