# Supplementary material for: Associations between multiple long-term conditions and mortality in diverse ethnic groups
Source: PLoS One. 2022 Apr 1;17(4):e0266418. doi: 10.1371/journal.pone.0266418 (PMC8974956; doi:10.1371/journal.pone.0266418)
Supplement: S6 Table — (DOCX) [file pone.0266418.s006.docx]

**S6 Table. Cox regression estimates for model including circulatory/endocrine/respiratory conditions**

|  | Circulatory condition | | | Endocrine condition | | | Respiratory condition | | |
| --- | --- | --- | --- | --- | --- | --- | --- | --- | --- |
| Covariate | Regression estimate | Standard error | p-value | Regression estimate | Standard error | p-value | Regression estimate | Standard error | p-value |
| Women | -0.27 | 0.01 | p<0.001 | -0.29 | 0.01 | p<0.001 | -0.29 | 0.01 | p<0.001 |
| Baseline age | 0.10 | 0.0007 | p<0.001 | 0.10 | 0.0006 | p<0.001 | 0.11 | 0.0005 | p<0.001 |
| Baseline condition present | 0.12 | 0.03 | p<0.001 | 0.43 | 0.03 | p<0.001 | 0.71 | 0.03 | p<0.001 |
| Age x condition present | 0.008 | 0.001 | p<0.001 | -0.005 | 0.001 | p<0.001 | -0.011 | 0.001 | p<0.001 |
| Ethnicity (main effect) |  |  |  |  |  |  |  |  |  |
| Bangladeshi | -0.26 | 0.22 | p=0.2 | -0.38 | 0.22 | p=0.1 | -0.03 | 0.19 | p=0.9 |
| Pakistani | -0.14 | 0.14 | p=0.3 | -0.25 | 0.16 | p=0.1 | 0.11 | 0.14 | p=0.4 |
| Indian | -0.53 | 0.12 | p<0.001 | -0.63 | 0.12 | p<0.001 | -0.43 | 0.11 | p<0.001 |
| Other Asian | -0.54 | 0.16 | p=0.001 | -0.46 | 0.16 | p=0.005 | -0.32 | 0.16 | p=0.04 |
| Chinese | -1.13 | 0.31 | p<0.001 | -1.14 | 0.32 | p<0.001 | -1.01 | 0.31 | p=0.001 |
| Black African | -0.26 | 0.13 | p=0.05 | -0.32 | 0.13 | p=0.02 | -0.01 | 0.12 | p=0.9 |
| Black Caribbean | 0.05 | 0.13 | p=0.7 | 0.08 | 0.13 | p=0.5 | 0.23 | 0.12 | p=0.07 |
| Other Black | -0.19 | 0.24 | p=0.4 | -0.13 | 0.23 | p=0.6 | 0.13 | 0.21 | p=0.5 |
| Mixed | 0.04 | 0.14 | p=0.8 | 0.06 | 0.13 | p=0.6 | 0.21 | 0.14 | p=0.1 |
| Other | -0.50 | 0.19 | p=0.007 | -0.36 | 0.19 | p=0.06 | -0.25 | 0.19 | p=0.2 |
| Age x ethnicity interaction: |  |  |  |  |  |  |  |  |  |
| Bangladeshi | -0.003 | 0.010 | p=0.8 | -0.007 | 0.010 | p=0.5 | -0.004 | 0.008 | p=0.6 |
| Pakistani | -0.014 | 0.006 | p=0.01 | -0.014 | 0.006 | p=0.02 | -0.010 | 0.005 | p=0.05 |
| Indian | 0.007 | 0.004 | p=0.1 | 0.007 | 0.004 | p=0.1 | 0.006 | 0.004 | p=0.2 |
| Other Asian | -0.006 | 0.008 | p=0.4 | -0.002 | 0.007 | p=0.7 | -0.003 | 0.007 | p=0.6 |
| Chinese | 0.017 | 0.012 | p=0.2 | 0.015 | 0.011 | p=0.2 | 0.019 | 0.010 | p=0.07 |
| Black African | -0.013 | 0.007 | p=0.06 | -0.019 | 0.007 | p=0.005 | -0.015 | 0.006 | p=0.02 |
| Black Caribbean | -0.014 | 0.005 | p=0.004 | -0.015 | 0.005 | p=0.003 | -0.013 | 0.004 | p=0.004 |
| Other Black | -0.041 | 0.013 | p=0.002 | -0.040 | 0.011 | p=0.002 | -0.027 | 0.010 | p=0.007 |
| Mixed | -0.020 | 0.007 | p=0.006 | -0.024 | 0.007 | p<0.001 | -0.022 | 0.006 | p<0.001 |
| Other | -0.006 | 0.008 | p=0.5 | -0.003 | 0.008 | p=0.9 | -0.002 | 0.007 | p=0.8 |
| Condition present x ethnicity interaction: |  |  |  |  |  |  |  |  |  |
| Bangladeshi | 0.23 | 0.34 | p=0.5 | 0.40 | 0.30 | p=0.2 | -0.28 | 0.31 | p=0.4 |
| Pakistani | 0.56 | 0.18 | p=0.002 | 0.67 | 0.15 | p<0.001 | 0.11 | 0.18 | p=0.5 |
| Indian | 0.10 | 0.12 | p=0.4 | 0.22 | 0.11 | p=0.04 | 0.04 | 0.12 | p=0.8 |
| Other Asian | 0.36 | 0.22 | p=0.1 | 0.10 | 0.19 | p=0.6 | -0.12 | 0.22 | p=0.6 |
| Chinese | 0.20 | 0.38 | p=0.6 | 0.46 | 0.28 | p=0.1 | 0.10 | 0.42 | p=0.8 |
| Black African | 0.15 | 0.20 | p=0.5 | 0.51 | 0.18 | p=0.005 | -0.80 | 0.34 | p=0.02 |
| Black Caribbean | 0.16 | 0.15 | p=0.3 | 0.14 | 0.13 | p=0.3 | -0.30 | 0.12 | p=0.01 |
| Other Black | 0.84 | 0.41 | p=0.04 | 0.96 | 0.31 | p=0.002 | 0.27 | 0.36 | p=0.4 |
| Mixed | 0.06 | 0.24 | p=0.8 | 0.16 | 0.24 | p=0.5 | -0.43 | 0.26 | p=0.09 |
| Other | 0.42 | 0.23 | p=0.06 | -0.02 | 0.21 | p=0.9 | -0.40 | 0.29 | p=0.2 |
