# Supplementary figures and images for: Associations between multiple long-term conditions and mortality in diverse ethnic groups
Source: PLoS One. 2022 Apr 1;17(4):e0266418. doi: 10.1371/journal.pone.0266418 (PMC8974956; doi:10.1371/journal.pone.0266418)

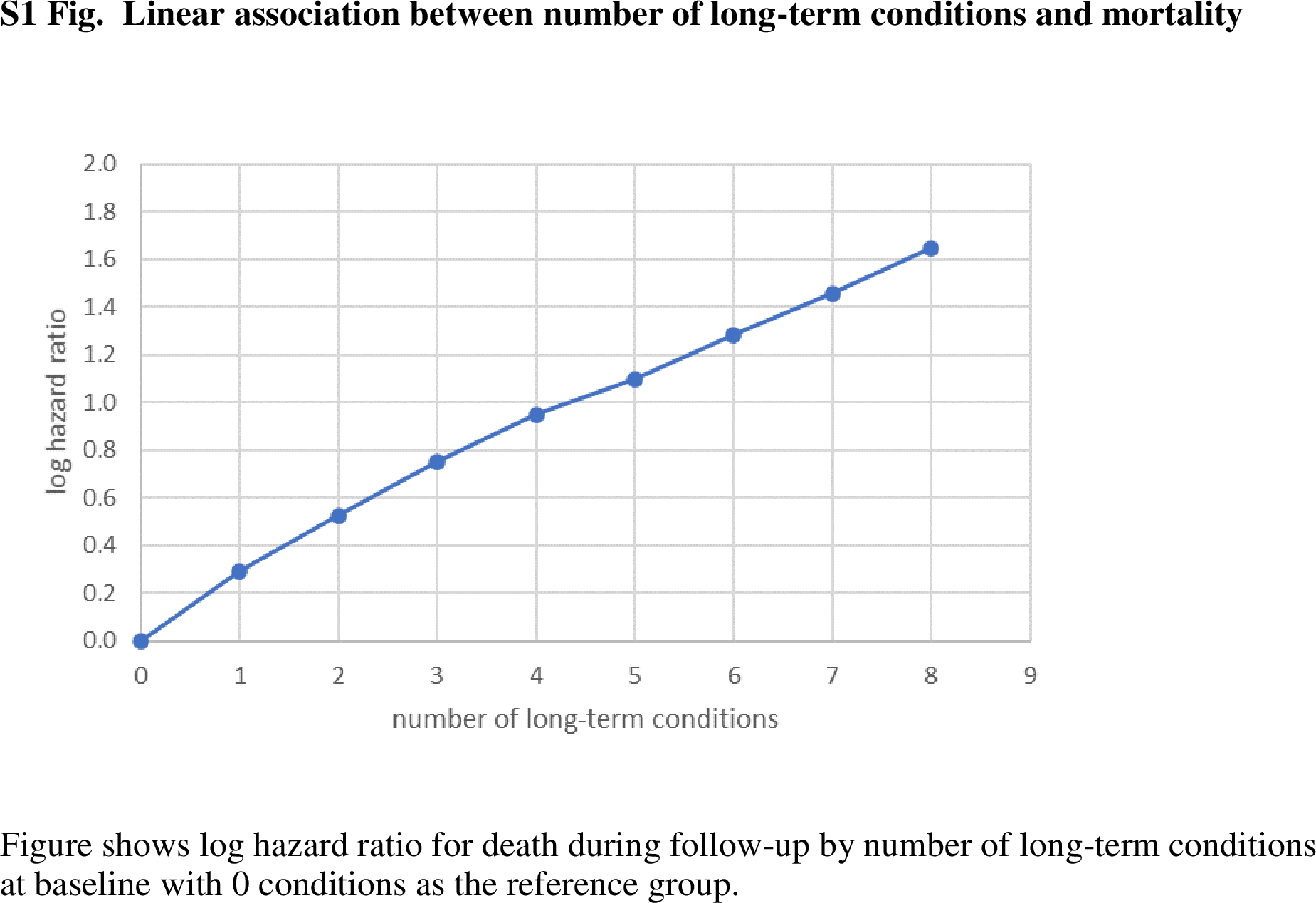

Supplement: S1 Fig — (TIF) [file pone.0266418.s007.tif]
